# Reduced Expression of ZDHHC2 Is Associated with Lymph Node Metastasis and Poor Prognosis in Gastric Adenocarcinoma

**DOI:** 10.1371/journal.pone.0056366

**Published:** 2013-02-15

**Authors:** Shu-Mei Yan, Jian-Jun Tang, Chun-Yu Huang, Shao-Yan Xi, Ma-Yan Huang, Jian-Zhong Liang, Yuan-Xue Jiang, Yu-Hong Li, Zhi-Wei Zhou, Ingemar Ernberg, Qiu-Liang Wu, Zi-Ming Du

**Affiliations:** 1 State Key Laboratory of Oncology in South China and Department of Pathology, Sun Yat-Sen University Cancer Center, Guangzhou, P. R. China; 2 Department of Gastric and Pancreatic Surgery, Sun Yat-Sen University Cancer Center, Guangzhou, P. R. China; 3 Department of Medical Oncology, Sun Yat-Sen University Cancer Center, Guangzhou, P. R. China; 4 Department of Experimental Research, Sun Yat-Sen University Cancer Center, Guangzhou, P. R. China; 5 Department of Microbiolgy, Tumor and Cell Biology, Karolinska Institutet, Stockholm, Sweden; Sapporo Medical University, Japan

## Abstract

**Background:**

Zinc finger, DHHC-type containing 2 (ZDHHC2), originally named as reduced expression associated with metastasis protein (REAM), has been proposed as a putative tumor/metastasis suppressor gene and is often aberrantly decreased in human cancers. However ZDHHC2 expression pattern and its clinical significance have not yet been investigated in gastric adenocarcinoma.

**Methodology/Principal Findings:**

Quantitative Real-Time PCR (qRT-PCR) and immunostaining were performed to detect ZDHHC2 expression in gastric adenocarcinoma, and then the correlation between ZDHHC2 expression and clinicpathologic parameters, and patient survival was analyzed. Compared to the adjacent normal tissues, ZDHHC2 expression was significantly reduced in gastric tumor tissues as shown by qRT-PCR and immunostaining. Low expression of ZDHHC2 was observed in 44.7% (211/472) of gastric adenocarcinoma patients, and was associated significantly with lymph node metastasis (p<0.001) and histological grade (p<0.001). Multivariate Cox regression analysis indicated that ZDHHC2 expression had a significant, independent predictive value for survival of gastric cancer patients (HR = 0.627, p = 0.001).

**Conclusions/Significance:**

Our data suggest that reduced ZDHHC2 expression is associated with lymph node metastasis and independently predicts an unfavorable prognosis in gastric adenocarcinoma patients.

## Introduction

Gastric cancer is the fourth most common malignancy and the second leading cause of cancer death in the world [1]. Although current treatment protocol for gastric cancer incorporates chemotherapy or radiation into surgical resection, the survival rate of gastric cancer patients remains poor [2]. The clinical stage at diagnosis and the options for curative surgery are the most important prognostic factors. However, regional lymph node metastasis, distant metastasis and loco-regional relapses frequently occur in spite of resection and multimodality therapy. Metastasis is the main cause of death from such tumors, but the mechanism of the metastatic process in gastric cancer is very complex and still not completely understood [3], [4], [5]. Hence novel well-characterized biomarkers would be helpful for clinicians to predict metastatic progression and prognosis of gastric cancer patients for facilitation of therapeutic intervention.

Protein palmitoylation refers to the posttranslational addition of a 16 carbon fatty acid to the side chain of cysteine, forming a thioester linkage, through the action of thiol-directed protein acyltransferases (PATs) [6], and this modification is readily reversible, providing a potential regulatory mechanism to mediate protein trafficking, organelle inheritance, and vesicle fusion [7], [8]. The PATs share a domain referred to as the DHHC domain, a cysteine-rich domain with a conserved aspartate-histidine-histidine-cysteine signature motif, which is directly involved in the palmitoyl transfer reaction [6]. There are at least 23 distinct mammalian DHHC proteins and eight yeast DHHC proteins, residing in diverse tissues and subcellular locations [9].

Zinc finger, DHHC-type containing 2 (ZDHHC2), one member of DHHC protein family of PATs, originally named as reduced expression associated with metastasis protein (REAM), is located in chromosome 8p21.3-22 [10], where frequent loss of heterozygosity has been detected in various types of metastatic cancers, including prostate cancer [11], hepatocellular carcinoma [12], colorectal cancer [13], non-small cell lung cancer [13], [14], urinary bladder [15], breast cancer [16], [17]. The mRNA level of ZDHHC2 expression was significantly reduced in primary and metastatic foci of advanced colorectal cancer [10].

The expression pattern of ZDHHC2 and its clinical significance in gastric adenocarcinoma have not been determined to date. Due to its proposed role in cancer, in this study, we aimed to investigate the expression pattern of ZDHHC2 in gastric adenocarcinoma, and its clinicopathological implications. Here we provided evidence that ZDHHC2 expression was significantly reduced in gastric tumor tissues, compared to the adjacent normal tissues. Reduction of ZDHHC2 expression was observed in 44.7% (211/472) of gastric adenocarcinoma patients, and was associated significantly with lymph node metastasis and histological grade. Furthermore, reduced ZDHHC2 expression is a significant, independent predictive factor for survival of gastric cancer patients.

## Materials and Methods

### Ethics Statement

The study was approved by the Ethics Committee of Sun Yat-sen University Cancer Center. All samples used in this study were anonymous and collected from patients for routine pathology use. No informed consent (written or verbal) was obtained for use of retrospective tissue samples from the patients in this study, since most of the patients were deceased and informed consent was not deemed necessary and waived by the Ethics Committee.

### Patients and Clinical Tissue Samples

For this retrospective study, archival formalin-fixed, paraffin-embedded (FFPE) tissue specimens from 472 primary gastric cancer patients who underwent surgical resection at Sun Yat-sen University Cancer Center from December 2002 to December 2006 were recruited. The patients who met the following eligibility criteria were included [18], [19]: (1) diagnosis of gastric adenocarcinoma identified by histopathological examination; (2) surgical history that included gastrectomy plus lymphadenectomy (limited or extended); (3) availability of complete follow-up data; (4) no preoperative treatment, such as chemotherapy and radiotherapy; (5) no history of familial malignancy or other synchronous malignancy (such as GIST, esophageal cancer, and colorectal cancer); (6) no recurrent gastric cancer and remnant gastric cancer; and (7) no death in the perioperative period. Tumor resection and D2 lymphadenectomy were performed by experienced surgeons, and the surgical procedures, which followed the Japanese Gastric Cancer Association (JGCA) guidelines [20], were similar in all patients who underwent radical resections. These patients included 325 male and 147 female patients, with a median age of 55 years (range, 17–85 years). Each tumor sample was assigned a histological grade based on the World Health Organization (WHO) classification criteria. All patients were staged using the 7th edition of the International Union Against Cancer (UICC) Tumor-Node-Metastasis (TNM) staging system.

For the qRT-PCR assay, fresh gastric cancer and paired adjacent non-tumor tissue samples were obtained from 45 gastric cancer patients who underwent surgical resection at the Sun Yat-sen University Cancer Center between 2011 and 2012. These 45 patients included 29 males and 16 females, with a median age of 57 years (range, 24–78 years). After surgical resection, the fresh tissue samples were immediately immersed in RNAlater (Ambion, Inc., USA) and stored at 4 degree overnight to allow thorough penetration of the tissues; the samples were then frozen at minus 80 degree until RNA extraction. Both the tumor tissue and the adjacent non-tumor tissue, which was located more than 2 cm away from the gastric cancer, were sampled and then verified by pathological examination.

### Immunohistochemistry

Immunohistochemistry (IHC) was performed as described previously [21]. ZDHHC2 antibody (1∶200 dilution, Cat#: AP5592a, Abgent, CA) was used. IHC results were evaluated and scored independently by two pathologists without knowledge of the clinicopathological outcomes of the patients.

Semiquantitative estimation was made using a composite score obtained by multiplying the values of staining intensity and relative abundance of positive cells. Intensity was graded as 0 (no staining), 1 (weak staining), 2 (moderate staining), or 3 (strong staining). The abundance of positive cells was graded from 0 to 4 (0, <5% positive cells; 1, 5–25%; 2, 26–50%; 3, 51–75%; 4, >75%). For Kaplan-Meier survival analysis, composite score greater than the median value was considered high expression, and composite score less than or equal to the median value was considered low expression. In addition we also validated these data by comparing the cases with highest composite score (score more than 8) with those with composite score (score less than 4).

### qRT-PCR Assays

For mRNA quantitive realtime PCR (qRT-PCR) assay, total RNA was extracted from gastric cancer tissues and adjacent normal tissues using TRIzol reagent (Invitrogen) according to the manufacturer’s protocol. RNAse-free DNAase I was used to eliminate DNA contamination. After reverse transcription of the total RNA, the first-strand cDNA was then used as template for detection of ZDHHC2 expression by qRT-PCR with the SYBR Green I chemistry (Power SYBR Green PCR Master Mix, CAT#: 4367659, ABI Inc., USA). GAPDH was used as internal control. The primers were ZDHHC2 (Forward: TCT TAG GCG AGC AGC CAA GGA T and Reverse: CAG TGA TGG CAG CGA TCT GGT T); GAPDH (Forward: AGC CAC ATC GCT CAG ACA C and Reverse: GCC CAA TAC GAC CAA ATC C). The relative expression level was determined as 2^−ΔΔCt^. Data are presented as the expression level relative to the calibrator (control sample).

### Statistical Analysis

Data was analyzed using SPSS12.0 software. A Chi-square test was used to determine the association between ZDHHC2 expression and clinicopathological parameters. Kaplan-Meier analysis and log-rank tests were used to assess the survival rate, and to compare differences in survival curves. Cox regression analysis was performed to assess the significance of multiple predictors of survival. Differences were considered significant at p<0.05.

## Results

### ZDHHC2 is Downregulated in Gastric Cancer

**Figure 1:**
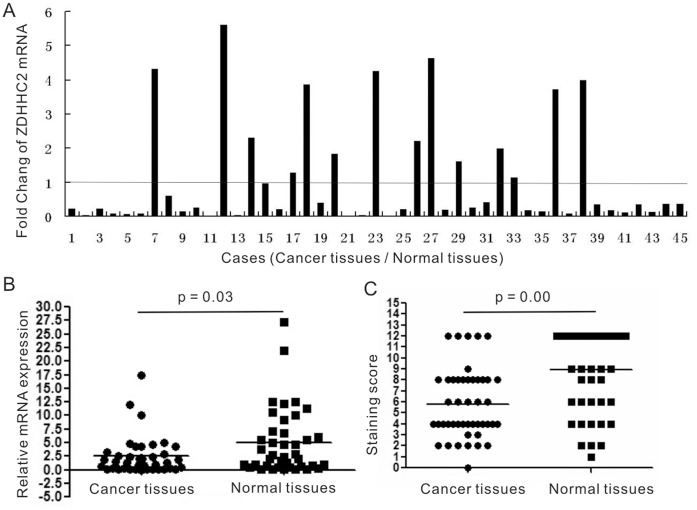
ZDHHC2 expression in gastric cancer and normal tissues. (**A**) The fold change of ZDHHC2 expression in gastric cancer tumor tissues compared to paired adjacent normal tissues (n = 45) assessed by qRT-PCR. (**B**)The average relative expression of mRNA level of ZDHHC2 in gastric cancer tumor tissues compared to paired adjacent normal tissues (n = 45). (**C**)The mean staining score of ZDHHC2 in gastric cancer tumor tissues compared to paired adjacent normal tissues (n = 45) assessed by immunohistochemistry.

To evaluate the difference in ZDHHC2 expression in gastric cancer and adjacent normal tissues, qRT-PCR was performed to detect ZDHHC2 expression in 45 cases of gastric cancer tissues and paired adjacent normal tissues. ZDHHC2 expression was reduced in 66.7% (30/45) of gastric cancer patients, compared with paired adjacent normal tissues (**Fig. 1A**). Furthermore, the average relative expression of ZDHHC2 in all 45 cases of gastric cancer tissues is lower than that of ZDHHC2 in adjacent normal tissues. There is significant difference in ZDHHC2 mRNA expression between cancer tissues and adjacent normal tissues (p = 0.03) (**Fig. 1B**).

**Figure 2:**
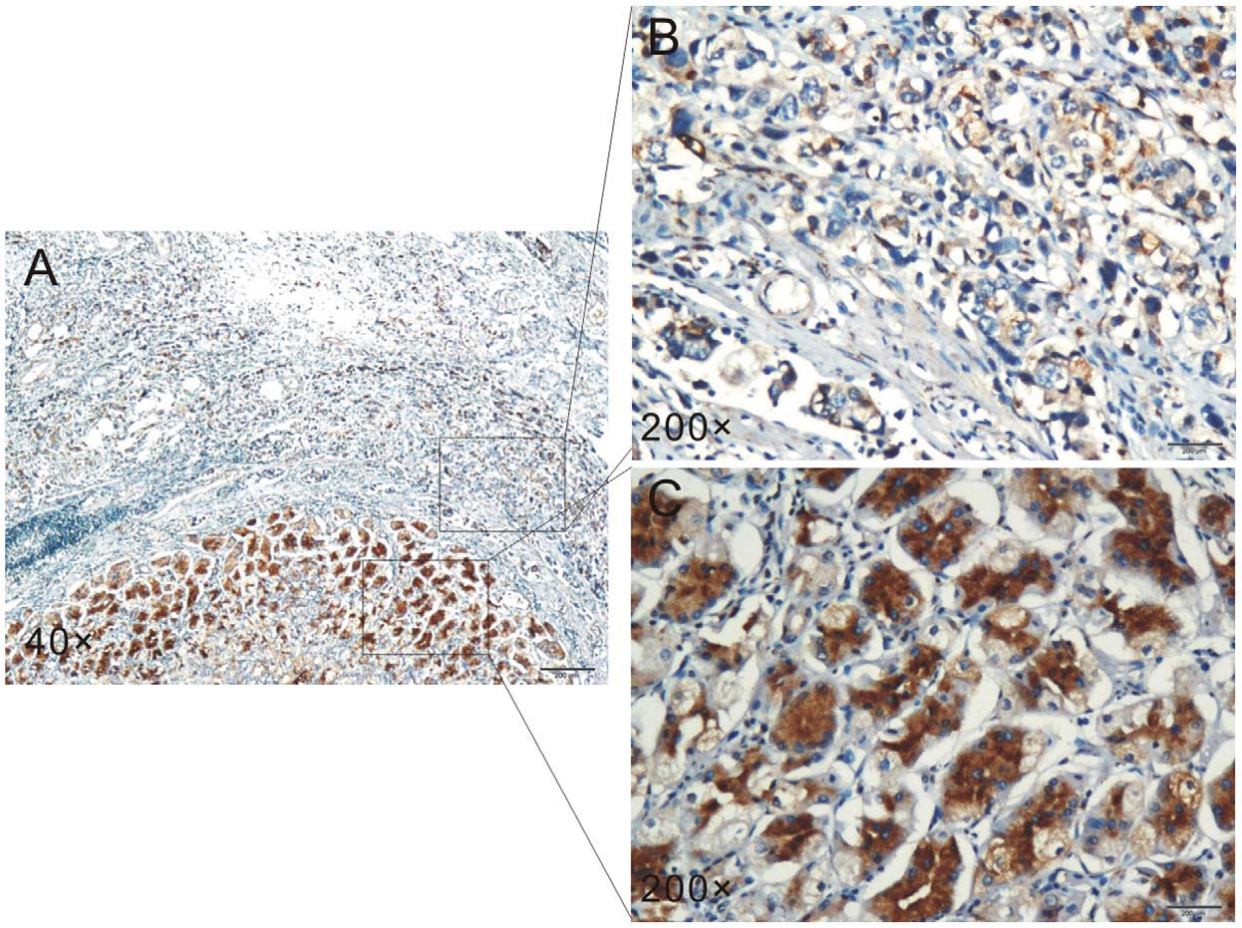
Immunohistochemistry analysis of ZDHHC2 expression in gastric cancer and adjacent normal tissues. Immunohistochemistry was performed to ZDHHC2 expression in gastric cancer tissues as well as adjacent normal tissues (**A**). Weak ZDHHC2 expression was observed in the cytoplasm of gastric cancer tissues (**B**). Strong ZDHHC2 expression was observed predominantly in the cytoplasm of adjacent normal tissues (**C**).

Moreover, immunohistochemistry was performed to detect ZDHHC2 expression in 45 cases of gastric cancer tissues and paired adjacent normal tissues. Semiquantitative estimation was made using a composite score obtained by multiplying the values of staining intensity and relative abundance of positive cells. ZDHHC2 expression was reduced in 68.9% (31/45) of gastric cancer patients as shown by immuohistochemistry, compared with paired adjacent normal tissues. Moreover, the mean staining score of ZDHHC2 in all 45 cases of gastric cancer tissues is lower than that of ZDHHC2 in adjacent normal tissues. There is significant difference in ZDHHC2 protein expression between cancer tissues and adjacent normal tissues (p = 0.00) (**Fig. 1C**). The representative figure of ZDHHC2 expression in gastric cancer tissues as well as adjacent normal tissues detected by immunohistochemistry was presented in **Fig. 2A**. Weak ZDHHC2 expression was observed in the cytoplasm of gastric cancer tissues (**Fig. 2B**), while strong ZDHHC2 expression was observed predominantly in the cytoplasm of adjacent normal tissues (**Fig. 2C**).

### Reduction of ZDHHC2 Expression Correlates with Clinicopathological Parameters

To investigate the association between ZDHHC2 expression and clinicopathological parameters of gastric cancer patients, paraffin-embedded tissues section (n = 472) with histopathologically confirmed gastric adenocarcinoma were examined using immunohistochemistry, and low expression of ZDHHC2 was observed in 44.7% (211/472) of gastric cancer patients.

**Figure 3:**
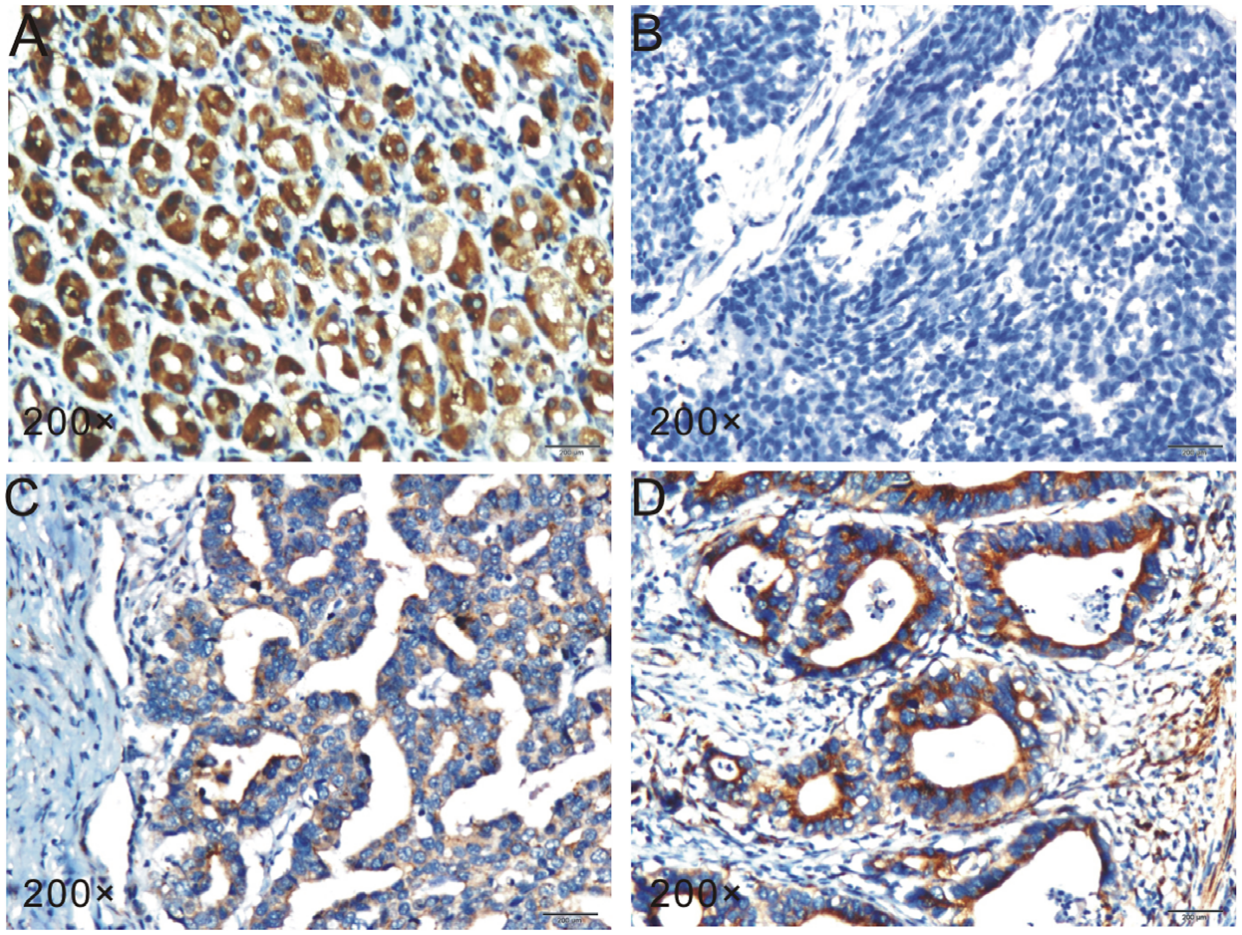
Immunohistochemistry analysis of ZDHHC2 expression in different differentiated gastric cancer. Compared to the normal tissues (**A**), ZDHHC2 expression was almost negative in patients with poorly differentiated carcinoma (G3) (**B**), while weak ZDHHC2 expression (G2) and strong ZDHHC2 expression (G1) were found in moderately differentiated gastric cancer and well-differentiated gastric cancer respectively (**C and D**).

Interestingly, reduced ZDHHC2 expression was associated significantly with lymph node metastasis (p<0.001) and histological grade (p<0.001) (**Table 1**). Compared to the normal tissues (**Fig. 3A**), ZDHHC2 expression was almost negative in patients with poorly differentiated carcinoma (G3) (**Fig. 3B**), while weak ZDHHC2 expression (G2) and strong ZDHHC2 expression (G1) were found in moderately differentiated gastric cancer and well-differentiated gastric cancer respectively (**Fig. 3C and 3D**). What is more, reduction of ZDHHC2 expression was associated significantly with lauren classification (p<0.001), tumor size (p = 0.026), TNM staging (p = 0.012) and location (p = 0.006). No significant association was seen between ZDHHC2 expression and age, gender, tumor infiltration, metastasis status or chemotherapy (**Table 1**).

**Table 1:** Correlation between ZDHHC2 expression and clinicopathological variables of 472 gastric cancer cases.

Parameters	Cases (n = 472)	ZDHHC2 expression		P value
		High expression	Low expression	
Age (years)				0.067
<55	193	97	96	
≥55	279	164	115	
Gender				0.797
Male	325	181	144	
Female	147	80	67	
Tumor size(cm)				0.026*
<3	63	43	20	
≥3	409	218	191	
Histological grade				<0.001*
Well differentiated (G1)	12	11	1	
Moderately differentiated (G2)	82	67	15	
Poorly differentiated (G3)	378	195	183	
Lauren classification				<0.001*
intestinal type	95	79	16	
diffuse type	289	124	165	
mix type	88	58	30	
Tumor infiltration				0.349
T1	33	22	11	
T2	44	27	17	
T3	92	54	38	
T4a	253	134	119	
T4b	50	24	26	
Nodal status (N)				<0.001*
N0	133	92	41	
N1	90	46	44	
N2	105	61	44	
N3	144	62	82	
Metastasis status (M)				0.118
M0	417	236	181	
M1	55	25	30	
TNM Staging				0.012*
I	50	37	13	
II	115	68	47	
III	252	131	121	
IV	55	25	30	
Location				0.006*
Proximal	270	151	119	
Distant	180	105	75	
Total	22	5	17	
Chemotherapy				0.495
No	191	102	89	
Yes	281	159	122	

* p<0.05, statistically significant.

### Reduction of ZDHHC2 Expression Predicts Poor Survival in Gastric Cancer

**Figure 4:**
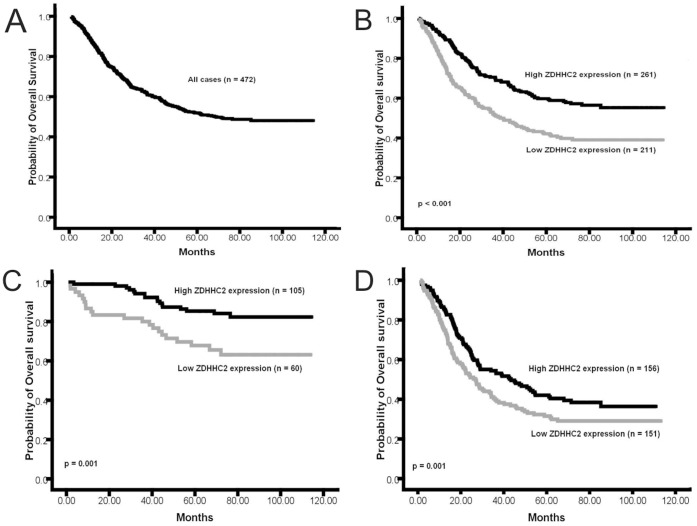
Kaplan-Meier curves for overall survival of the 472 gastric cancer patients. (**A**), Kaplan-Meier curves for overall survival (OS) of the 472 gastric cancer patients; (**B**), Kaplan-Meier curves for OS in gastric cancer patients with low level and high level ZDHHC2 expression; (**C**), Kaplan-Meier curves for OS in early stage (stage I and II) gastric cancer patients with low level and high level ZDHHC2 expression; (**D**), Kaplan-Meier curves for OS in advanced stage (stage III and IV) gastric cancer patients with low level and high level ZDHHC2 expression.

To investigate the prognostic value of ZDHHC2 expression in gastric cancer patients, overall survival (OS) analysis was performed in these 472 gastric cancer cases, and the five-year OS rate was 51.9% for these patients (**Fig. 4A**). The five-year OS rate was 41.6% for patients with low ZDHHC2 expression (n = 211), and 59.3% for patients with high ZDHHC2 expression (n = 261), which was a significant difference (p<0.001, **Fig. 4B**). In early stage (stage I and II) gastric cancer, the patients with the low levels of ZDHHC2 expression (n = 60) had a poorer prognosis than the patients with high levels of ZDHHC2 expression (n = 105) (p = 0.001, **Fig. 4C**). Meanwhile, in late stage (stage III and IV) gastric cancer, the patients with the low levels of ZDHHC2 expression (n = 151) also had a poorer prognosis than the patients with high levels of ZDHHC2 expression (n = 156) (p = 0.001, **Fig. 4D**).

**Figure 5:**
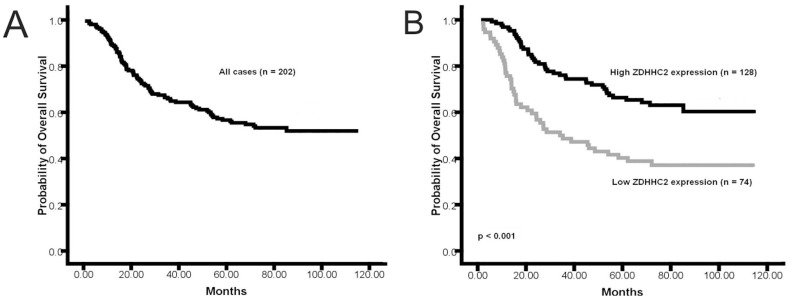
Kaplan-Meier curves for overall survival of 202 gastric cancer patients with highest ZDHHC2 expression and with lowest ZDHHC2 expression. (**A**), Kaplan-Meier curves for overall survival (OS) of the 202 gastric cancer patients; (**B**), Kaplan-Meier curves for OS in gastric cancer patients with highest ZDHHC2 expression (composite score more than 8, n = 128) and with lowest ZDHHC2 expression (composite score less than 4, n = 74).

To avoid the uncertainty of ZDHHC2 score around the median, we also compared the five-year OS rate of the gastric cancer patients with lowest ZDHHC2 expression (composite score less than 4, n = 74) with those with highest ZDHHC2 expression (composite score more than 8, n = 128). The five-year OS rate was 38.9% for patients with lowest ZDHHC2 expression, and 65.3% for patients with highest ZDHHC2 expression. The patients with the highest ZDHHC2 expression had a much better prognosis than the patients with lowest levels of ZDHHC2 expression (p<0.001, **Fig. 5**).

Univariate and multivariate analyses were performed to compare the impact of ZDHHC2 expression and other clinicopathological parameters on prognosis. Univariate analyses showed that 11 factors, including ZDHHC2 expression (p<0.001), metastasis status (p<0.001), nodal status (p<0.001), TNM staging (p<0.001), tumor infiltration (p<0.001), location (p = 0.001), lauren classification (p = 0.04), histological grade (p = 0.007), tumor size (p<0.001), age (p = 0.003) and chemotherapy (p<0.001) were prognostic predictors of OS in gastric cancer patients. Then all 11 factors were included in a multivariate Cox proportional hazards model to adjust for the effects of covariates. Based on this model, the relative risk of death in patients with high ZDHHC2 expression tumors was lower than that of patients with low ZDHHC2 expression tumors (HR = 0.627, 95% CI = 0.476–0.826). ZDHHC2 expression had a significant, independent predictive value for survival of gastric cancer patients (p = 0.001). Moreover tumor infiltration (p = 0.002), nodal status (p = 0.014), metastasis status (p = 0.013), age (p = 0.002), location (p = 0.001) and chemotherapy (p<0.001) were also independent prognosis predictors for gastric cancer patients (**Table 2**).

**Table 2:** Univariate and multivariate analyses of overall survival of gastric cancer patients.

Variables	Univariate analyses			Multivariate analyses		
	HR	(95%CI)	p value	HR	(95%CI)	p value
Age (years),(<55 vs. ≥55)	1.508	1.149–1.977	0.003*	1.585	1.190–2.111	0.002*
Gender (male vs. female)	1.066	0.808–1.406	0.65			
Tumor size(cm),(<3 vs. ≥3)	3.492	1.995–6.114	<0.001*	1.327	0.731–2.410	0.353
Histological grade, (G1/G2/G3)	1.536	1.123–2.102	0.007*	1.197	0.710–2.016	0.500
lauren classification, (intestinal/mix/diffuse type)	1.237	1.010–1.515	0.04*	0.905	0.650–1.258	0.552
Tumor infiltration (T1/T2/T3/T4a/T4b)	1.813	1.550–2.121	<0.001*	1.368	1.118–1.674	0.002*
Nodal status (N),(N0/N1/N2/N3)	1.553	1.384–1.743	<0.001*	1.232	1.043–1.454	0.014*
Metastasis status (M),(M0/M1)	5.285	3.819–7.314	<0.001*	2.349	1.201–4.594	0.013*
TNM Staging, (I/II/III/IV)	3.226	2.620–3.972	<0.001*	1.595	0.986–2.581	0.057
ZDHHC2 expression, (Low/High)	0.575	0.444–0.744	<0.001*	0.627	0.476–0.826	0.001*
Location,(Proximal/Distant/Total)	0.657	0.513–0.841	0.001*	0.656	0.515–0.835	0.001*
Chemotherapy, (No vs. Yes)	0.384	0.296–0.498	<0.001*	0.367	0.282–0.479	<0.001*

* p<0.05, statistically significant.

## Discussion

ZDHHC2, one member of ZDHHC family, originally named as reduced expression associated with metastasis protein (REAM), has been proposed as a putative tumor/metastasis suppressor gene, and the mRNA level of ZDHHC2 expression was found to be significantly reduced in primary and metastatic foci of advanced colorectal cancer [10], However, ZDHHC2 expression pattern has not yet been investigated in gastric adenocarcinoma. To our knowledge, this is the first report that ZDHHC2 expression was significantly reduced in gastric tumor tissues compared to the adjacent normal tissues as shown by qRT-PCR and immunostaining. Reduction of ZDHHC2 expression was observed in 44.7% (211/472) of gastric adenocarcinoma patients, and was associated significantly with lymph node metastasis (p<0.001) and histological grade (p<0.001). Furthermore, our results indicated that ZDHHC2 expression had a significant, independent predictive value for survival of gastric cancer patients (p = 0.001). Hence, our results also proposed that ZDHHC2 was a putative tumor/metastasis suppressor in gastric adenocarcinoma, which were consistent with previous studies of ZDHHC2.

Tumor progression is regulated precisely by a small subset of genes that act by either activation of oncogenes or silence of tumor suppressor genes [22]. Tumor suppressor gene could negatively regulate cell proliferation, namely operate in various ways to limit cell growth and proliferation. In cancer cells, tumor suppressor genes are usually silenced by genetic alteration [23] or epigenetic alteration [24]. ZDHHC2 is located in chromosome 8p21.3-22 [10], where frequent loss of heterozygosity has been detected in various types of metastatic cancers [11], [12], [13], [14], [15], [16], [17], and somatic mutations of this gene were found in colorectal cancer, hepatocellular carcinoma, and nonsmall lung cancer [10]. Haploid insufficiency has been considered to be one of the mechanisms for loss of tumor suppressive function of some genes in experimental cancer models [25], [26]. Therefore we proposed that the reduction of ZDHHC2 expression in gastric adenocarcinoma might be caused by several different ways, including haploid insufficiency due to loss of one chromosome, mutation and epigenetic alteration. Moreover, microarray data in Giannakis M et al’ s study [27] suggested that helicobacter pylori infection might contribute to downregulation of ZDHHC2 in gastric cancer.

In the present study, reduced ZDHHC2 expression in gastric cancer was found to be associated significantly with lymph node metastasis (p<0.001), suggesting that ZDHHC2 might play an important role in gastric cancer metastasis. However, in our study, no significant correlation was found between ZDHHC2 expression and distant tumor metastasis in gastric adenocarcinoma. It might be because of the low number of gastric patients with distant metastasis in our study, since gastric patients with metastasis usually give up surgical resection. The multistep process of invasion and metastasis has been schematized as a sequence of discrete steps, often termed the invasion-metastasis cascade [28]. This process includes several steps, beginning with local invasion, then intravasation by cancer cells into nearby blood and lymphatic vessels, transit of cancer cells through the lymphatic and hematogenous systems, followed by escape of cancer cells from the lumina of such vessels into the parenchyma of distant tissues (extravasation), the formation of small nodules of cancer cells (micrometastases), and finally the growth of micrometastatic lesions into macroscopic tumors, this last step being termed “colonization.” [29], [30]. As ZDHHC2 is one of PATs, which is directly involved in the palmitoyl transfer reaction [31], hence identification of a hypo/depalmitoylated substrate of ZDHHC2 would provide important insight into the molecular mechanisms underlying metastasis.

Many substrate have already been identified for ZDHHC2, including the neuronal adaptor/scaffold protein PSD95 [32], the SNARE proteins SNAP-23/25 [33], nudE nuclear distribution E homolog -like 1 (NDEL1) [34], the transmembrane proteins CD9 and CD151 of the tetraspanin family [35], cytoskeleton-associated protein 4 (CKAP4) [36], ATP-binding cassette transporter A1 (ABCA1) [37], lymphocyte-specific protein tyrosine kinase (Lck) [38], G protein alpha subunit [39], regulator of G-protein signaling 7 binding protein (R7BP) [40] and endothelial nitric oxide synthase (eNOS) [41]. However there is no apparent structural similarity between the reported substrates of ZDHHC2, or even any sequence similarities surrounding the residues being S-acylated. ZDHHC2 can mediate S-acylation of cysteines located in the N terminal region (PSD-95 and G protein alpha subunit), internally in the protein sequence (SNAP-23/25), in the juxtamembrane region of transmembrane proteins (CD9 and CD151) and close to an N-terminal myristoylated glycine (Lck and eNOS) [38].

Which substrate could make ZDHHC2 function as putative tumor/metastasis suppressor? The first substrate of ZDHHC2 related to its tumor/metastasis suppressor function is CKAP4/p63 [36], which was identified as a cell surface receptor for antiproliferative factor (APF) [42]. APF profoundly inhibits cellular proliferation and induces well-characterized, specific changes in the expression of genes involved in cell migration and adhesion, with a concomitant change in the phenotype of the cells toward a more differentiated state [43], [44]. These changes in cellular behavior are mediated through high-affinity binding of APF to CKAP4, as CKAP4 gene knockdown and immunodepletion abrogate APF signaling [42]. The discovery that CKAP4/p63, the receptor for APF, is a substrate of ZDHHC2, provides an important link between the proliferative properties of many types of cancer cells and the reduced expression or mutation of ZDHHC2 [36]. The second substrate of ZDHHC2 related to its putative tumor/metastasis suppressor is tetraspanins CD9 and CD151 [35]. ZDHHC2 could palmitoylate the tetraspanins CD9 and CD151, promoting physical associations between them and protecting them from lysosomal degradation, while a 70% reduction in ZDHHC2 mRNA expression by siRNA mediated knockdown resulted in lysosomal targeting and rapid degradation of CD9. [35]. This discovery provides one plausible mechanism by which ZDHHC2 may function as a tumor suppressor. It is possible that hypopalmitoylation of both CKAP4 and CD9 may increase tumor or metastatic behavior. In either case, the importance of maintaining ZDHHC2 expression to suppress metastatic cellular behavior is becoming clearer. Hence, the substrate and function of ZDHHC2 in gastric cancer deserve for further study.

In conclusion, reduced ZDHHC2 in gastric adenocarcinoma is associated with lymph node metastasis and poor prognosis of the patients. The potential of ZDHHC2 as therapeutic targets for gastric adenocarcinoma should be further investigated.
